# *Colletotrichum higginsianum* Mitogen-Activated Protein Kinase ChMK1: Role in Growth, Cell Wall Integrity, Colony Melanization, and Pathogenicity

**DOI:** 10.3389/fmicb.2016.01212

**Published:** 2016-08-03

**Authors:** Wei Wei, Ying Xiong, Wenjun Zhu, Nancong Wang, Guogen Yang, Fang Peng

**Affiliations:** ^1^Institute for Interdisciplinary Research, Jianghan UniversityWuhan, China; ^2^Hefei Inzyme Information Technology Co., Ltd.Wuhan, China; ^3^College of Biology and Pharmaceutical Engineering, Wuhan Polytechnic UniversityWuhan, China; ^4^The Provincial Key Lab of Plant Pathology of Hubei Province, College of Plant Science and Technology, Huazhong Agricultural UniversityWuhan, China

**Keywords:** *Colletotrichum higginsianum*, MAPK, pathogenicity, cell wall integrity, growth rate

## Abstract

*Colletotrichum higginsianum* is an economically important pathogen that causes anthracnose disease in a wide range of cruciferous crops. To facilitate the efficient control of anthracnose disease, it will be important to understand the mechanism by which the cruciferous crops and *C. higginsianum* interact. A key step in understanding this interaction is characterizing the mitogen-activated protein kinases (MAPK) signaling pathway of *C. higginsianum*. MAPK plays important roles in diverse physiological processes of multiple pathogens. In this study, a Fus3/Kss1-related MAPK gene, *ChMK1*, from *C. higginsianum* was analyzed. The results showed that the Fus3/Kss1-related MAPK ChMK1 plays a significant role in cell wall integrity. Targeted deletion of *ChMK1* resulted in a hypersensitivity to cell wall inhibitors, reduced conidiation and albinistic colonies. Further, the deletion mutant was also unable to form melanized appressorium, a specialized infection structure that is necessary for successful infection. Therefore, the deletion mutant loses pathogenicity on *A. thaliana* leaves, demonstrating that *ChMK1* plays an essential role in the early infection step. In addition, the *ChMK1* deletion mutant showed an attenuated growth rate that is different from that of its homolog in *Colletotrichum lagenarium*, indicating the diverse roles that Fus3/Kss1-related MAPKs plays in phytopathogenic fungi. Furthermore, the expression level of three melanin synthesis associated genes were clearly decreased in the albinistic *ChMK1* mutant compared to that of the wild type strain, suggesting that ChMK1 is also required for colony melanization in *C. higginsianum*.

## Introduction

The hemibiotrophic fungal pathogen *Colletotrichum higginsianum* causes anthracnose disease. The disease typically manifests as small, dark, discrete spots or water-soaked, sunken lesions on the leaves, stems, or fruits of a wide range of cruciferous plants, including the model plant *Arabidopsis thaliana* (Narusaka et al., 2004; O'Connell et al., 2004; Birker et al., 2009; Ushimaru et al., 2010). *C. higginsianum* has emerged as an attractive model for studying both fungal pathogenicity and plant immune responses due to the availability of genomic and transcriptomic databases (O'Connell et al., 2012; Gan et al., 2013), and the ease of genetic manipulation of both the host plant and the pathogen (O'Connell et al., 2004; Zhang et al., 2006; Huser et al., 2009; Narusaka et al., 2009).

By recognizing host physical and chemical cues, *C. higginsianum* conidia differentiate melanized appressorium, an infection structure, at the tips of conidial germ tubes. Appressorium formation is required for successful infection since the fungus penetrates the cuticle and plant cell wall by utilization of enormous turgor pressure in melanized appressoria for further invasive growth (O'Connell et al., 2012). Thus, inhibition of melanized appressorium formation will facilitate the efficient control of anthracnose disease.

The infection-related morphogenesis and invasive growth of several appressorium-forming pathogens are regulated by many signal transduction pathways, especially the mitogen-activated protein kinase (MAPK) pathway (Xu and Hamer, 1996; Lev et al., 1999; Takano et al., 2000; Kojima et al., 2002; Bruno et al., 2004). To date, the MAPK cascades and MAPK signaling pathways are known to be involved in many major cell processes in fungi, such as stress responses, vegetative growth, pathogenicity, secondary metabolism, and cell wall integrity (Zhao et al., 2007; Turrà et al., 2014; Qi et al., 2015). CoMEKK1, a homolog of MAPKKK STE11, the up-stream regulator of Fus3/Kss1-related MAPK pathway, is essential for appressorium development and pathogenicity in *C. orbiculare* (Sakaguchi et al., 2010). Moreover, the CoIra1 in *C. orbiculare* is also involved in infection-related conidial germination and appressorium formation by proper regulation of Fus3/Kss1-related MAPK signaling pathways through CoRas2 on the up-stream (Harata and Kubo, 2014). In *Magnaporthe oryzae*, phosphodiesterase MoPdeH interacts with MoMck1, and functions upstream of the MoMck1–MoMkk1–MoMps1 MAPK pathway to regulate cell wall integrity (Yin et al., 2016). *M. oryzae* TRX2 interacts with Mst7, thus regulating the activation of Pmk1 MAPK via the Mst11-Mst7-Pmk1 MAPK pathway. Deletion of the *TRX2* gene caused pleiotropic defects in conidiation, growth, responses to stresses, and plant infection progression (Zhang et al., 2016). By phosphorylation on MAPK Fmk1, *Fusarium oxysporum* Fbp1 regulates virulence, cell wall integrity, and invasive growth via the Fmk1 signal pathway (Miguel-Rojas and Hera, 2016). MAPKs related to the yeast Slt2, such as SLT2-type MAPK protein PsMPK1, and PsMPK7 from *Phytophthora sojae* are also important for hyphal growth, cell wall integrity, stress tolerance, ROS detoxification, and pathogenicity (Li et al., 2014; Gao et al., 2015). Mutation of three MAPK genes *FoSlt2, FoMkk2*, and FoBck1 respectively led to attenuated virulence and slower growth rate in *F. oxysporum* (Ding et al., 2015). The MAPK AaSLT2 in *Alternaria alternate* regulates conidiation, virulence, and melanin production (Yago et al., 2011). Therefore, these findings suggest significant roles for MAPK signaling pathways in multiple physiological processes of different microorganisms; inhibition on the MAPK signaling pathway of pathogens will disturb infection progresses and facilitate the efficient control of crop disease.

Although many studies have examined MAPK signaling pathways in other fungi, functional analysis in *C. higginsianum* is still required to understand the intricate roles in the *A. thaliana*–*C. higginsianum* interaction. To date, compared with other fungi, the specific roles of MAPK for infection-related morphogenesis remain largely unknown in *C. higginsianum*. Because the MAPK pathway contributes to multiple physiological processes of fungal pathogens (Zhang et al., 2016), characterization of *C. higginsianum* MAPK will help illuminate the mechanism of the cruciferous crops—*C. higginsianum* interaction and facilitate the efficient control of anthracnose disease. For characterizing the MAPK involved in appressorium formation and pathogenicity ability of *C. higginsianum*, we here investigated the functions of ChMK1 (CH063_08490), an ortholog of PMK1 involved in appressorium differentiation and plant infection in *M. oryzae* (Bruno et al., 2004). Besides attenuated growth rate, reduced conidiation and albinistic colony, our results firstly indicated that targeted disruption of Fus3/Kss1-related MAPK gene *ChMK1* leads to hypersensitivity to cell wall inhibitors. Moreover, the *ChMK1* gene disruption mutant also failed to form appressorium and lost its pathogenicity. Here, we have reported the roles of ChMK1 in cell wall integrity, appressorium differentiation, and pathogenicity in *C. higginsianum*, and demonstrated that the MAPK signaling pathways play essential roles in this fungus.

## Materials and methods

### Fungal strains, plants, and culture condition

The wild-type *C. higginsianum* IMI349061 (CH-1) was cultured on potato dextrose agar (PDA) at 25°C and stored in PDA slants at 4°C for further use. *A. thaliana* wild-type Col-0 plants were used in this study. Plants were grown in growth chambers at day and night temperatures of 20 ± 2°C, with 12 h of light and 12 h of darkness. *Escherichia coli* strain DH5α was used to propagate all plasmids and *Agrobacterium tumefaciems* strain EHA105 was used for fungal transformation.

### Bioinformatics data and programs used in this study

The publicly available genomic sequence database of *C. higginsianum* (http://genome.jgi.doe.gov/Colhi1/Colhi1.home.html) was used to characterize the *CH063_08490* gene. *NCBI* (http://www.ncbi.nlm.nih.gov/) and *UniProt* (http://www.uniprot.org/blast/) were used for Blastp analysis. The *Clustal X* program was used for amino acid alignments. *MEGA* program was used to produce the phylogenetic tree with unrooted neighbor-joining method. The secondary structure prediction was completed with *Jnetpred* program.

### *ChMK1* gene replacement and complementary

For characterizing the *ChMK1* gene, a *ChMK1* replacement vector, pChMK1-3300, and a complementation vector, pChMK1-Com, were constructed. The replacement vector was constructed using the homologous recombination strategy. A 0.9-kb fragment upstream of the *ChMK1* ORF in the *C. higginsianum* genome was amplified with primers Rep-up F and Rep-up R (Table 1) and cloned into the *Hind* III and *Sal* I sites on pMD18-*hph*, and the resulting construct was named pMD2-*hph*. Then a 1.0-kb fragment downstream of *ChMK1* ORF was amplified with primers Rep-down F and Rep-down R (Table 1) and cloned between the *Xba* I and *Kpn* I sites in pMD2-*hph*, and the resulting construct was the *ChMK1* gene replacement vector, pChMK1-3300 (Figure S1), which had the selective marker *hph* gene flanked by the *ChMK1* ORF flanking sequences (**Figure 2A**).

**Table 1:** **Primers used for vector construction and PCR**.

**Primers used for vector construction and PCR**	
*ChMK1* replacement vector for upstream	Rep-up F: 5′ AAGCTTCCAAAACTTATCGGGGGC 3′
	Rep-up R: 5′ GTCGACATTGTTGGCGATGTGCG 3′
*ChMK1* replacement vector for downstream	Rep-down F: 5′ TCTAGAGCCCTCAAACACCCTTACC 3′
	Rep-down R: 5′ GGTACCGGGCAACGACGACACAAA 3′
*ChMK1* complementary vector	Com F: 5′ CCCGGGATGTCGCGCGCGAACCCCCCC 3′
	Com R: 5′ CTGCAGTCACCGCATGATCTCCTGGTAGATC 3′
*ChMK1* for RT-PCR and qRT-PCR	RT F: 5′ CAAGACCTATCCGACGACCACT 3′
	RT R: 5′ CAAGACCTATCCGACGACCACT 3′
*PKS1* for qRT-PCR	*PKS1* F: 5′ AACTGTCCACCACATCCATTCACGC 3′
	*PKS1* R: 5′ AGAGGGTAGAAGGGCACAGAGGAGC 3′
*THR1* for qRT-PCR	*THR1* F: 5′ GTCGCCCGCGAGGCCTACAAGAACC 3′
	*THR1* R: 5′ ACCAAAGTCGATGGCCATGCAGCGG 3′
*SCD1* for qRT-PCR	*SCD1* F: 5′ CTGGGACCGCCTCCGCAAGTGCATT 3′
	*SCD1* R: 5′ GAGACCTTCTCGTAGCGGGTGCCGC 3′
β-*tubulin* for qRT-PCR reference gene	β-*tubulin* F: 5′ AGAAAGCCTTGCGACGGAACA 3′
	β-*tubulin* R: 5′ CCTCCAGGGTTTCCAGATTA 3′

The complementation vector was constructed that *ChMK1* cDNA was amplified by RT-PCR with primers Com F and Com R (Table 1) and cloned into the same sites of pCIT vector, which contained the constitutive P*trpC* promoter and terminator. Finally, the cDNA of *ChMK1* was digested with *Apa* I and cloned into pNeoP3300, resulting in *ChMK1* complementation vector, pChMK1-Com.

*Agrobacterium*-mediated transformation was performed as previously described (Liu et al., 2013) with some modification that plasmid-containing *A. tumefaciems* cultures (0.6 OD unit at 600 nm) were mixed 1:1 with *C. higginsianum* conidial suspension (10^6^ spores/ml) in induction broth supplemented with 400 μM acetosyringone and cultured for 6 h at 25°C 200 rpm, and then cultured on a cellophane membrane laid on co-induction medium supplemented with 400 μM acetosyringone at 22°C for 48 h. The cellophane membrane were removed to new plates and overlaid with 15 ml PDA containing 500 μg/ml of cephalosporin and 50 μg/ml of hygromycin. After incubation at 25°C for 72 h, transformants were transferred to PDA plates containing 50 μg/ml of hygromycin for a second round of selection. Transformants were confirmed primarily by RT-PCR with primers RT F and RT R (Table 1) and further confirmed by Southern blot. The *C. higginsianum* β*-tubulin* gene (CH063_04743) (Table 1) was used to normalize the RNA sample for each RT-PCR.

### DNA manipulation and southern blot analysis

The genomic DNA of *C. higginsianum* wild-type strain and other derivative mutants was extracted according to the procedure (Sambrook and Russell, 2001).

Southern blot analysis was performed following the method described (Liu et al., 2013). For each sample, 15–20 μg genomic DNA was digested with *Hind* III, size-fractionated through a 0.8% agarose gel and mounted on positively charged nylon membrane. The nylon membrane was then hybridized with a probe amplified by primers RT-F and RT-R (Table 1) and labeled with digoxigenin (DIG)-dUTP using the PCR DIG Probe Synthesis Kit (Roche, Mannheim, Germany) in accordance with the manufacturer's instructions.

### RNA manipulation and qRT-PCR

Total RNA of fungal strains was isolated with TriZOL reagent (Invitrogen, Carlsbad, USA) according to manufacturer's instructions and stored at −80°C for further study. The total RNA samples were treated with DNase I (RNase Free) (Takara, Dalian, China) at 37°C for 0.5 h and used to generate the first strand cDNA with RevertAid™ First Strand cDNA Synthesis Kit (Fermentas, St. Leon-Rot, Germany) according to manufacturer's instructions. The first strand cDNA was stored at −20°C for further study.

Gene expression was analyzed by qRT-PCR using a Bio-Rad CFX96 (Bio-Rad, USA) and SYBR Green PCR mix (Bio-Rad, USA), according to the manufacturer's instructions. The *C. higginsianum* β*-tubulin* gene (CH063_04743) was used to normalize the RNA sample for each qRT-PCR. For each gene, qRT-PCR assays were repeated at least twice, with each repetition having three replicates.

Primer pairs for qRT-PCR detections were designed across or flanking an intron and listed in Table 1.

### Characterization of ChMK1 deletion transformant and wild-type strain

The growth rates were assayed that *ChMK1* deletion transformant and the wild-type strain were firstly cultivated on PDA for 7 days, then the mycelial agar discs were taken from the active colony edge and inoculated on the center of the PDA petri dish at 25°C before hyphal growth was examined. The colony morphology and conidiation of these strains were examined after being grown on PDA plate for 15 days at 25°C.

The virulence was assayed that *C. higginsianum* strains were cultured on PDA at 25°C for 5 days, and the conidia were collected and washed with sterile distilled water twice. The conidial suspension (10^6^ conidia/ml) were spotted with 6 μl droplets on the leaf surface of 4-weeks-old Arabidopsis. Inoculated leaves were incubated in darkness at 25°C, and the symptoms were observed at 6 days post-inoculation (dpi).

Conidial germination and appressorial formation were observed that 10 μl conidial suspension (10^6^ conidia/ml) was spotted on plastic coverslips and incubated at 25°C for 24 h. Then, the infection-related morphogenesis was examined by Nikon Eclipse 80i microscope (Nikon, Tokyo, Japan), under bright-field model using 40 × fold magnification.

### Cell wall sensitivity assay

The sensitivity of wild-type strain and the transformants to cell wall inhibitors were performed that mycelial plugs (4 mm in diameter) were respectively inoculated onto PDA containing 0.01% SDS, 300 μg/ml Calcofluor White (CFW), and 300 μg/ml Congo Red (CR) for 7 days as described (Zeng et al., 2012; Luo et al., 2014), and with PDA media as controls. Cell wall sensitivity to the compounds mentioned above was assayed by measuring the colony diameters as previously described (Fujioka et al., 2007; Valiante et al., 2009; Carbo and Perez-Martin, 2010).

### Statistical analysis

The data assays were analyzed with Origin 7.5 (OriginLab Corporation, Massachusetts, USA) using ANOVA (one-way, *P* ≤ 0.05). Results of all graphs represent the mean value ± SD. Asterisks in the graphs indicate statistical differences, *P* ≤ 0.05.

## Results

### CH063_08490 is similar to MAPK protein

**Figure 1:**
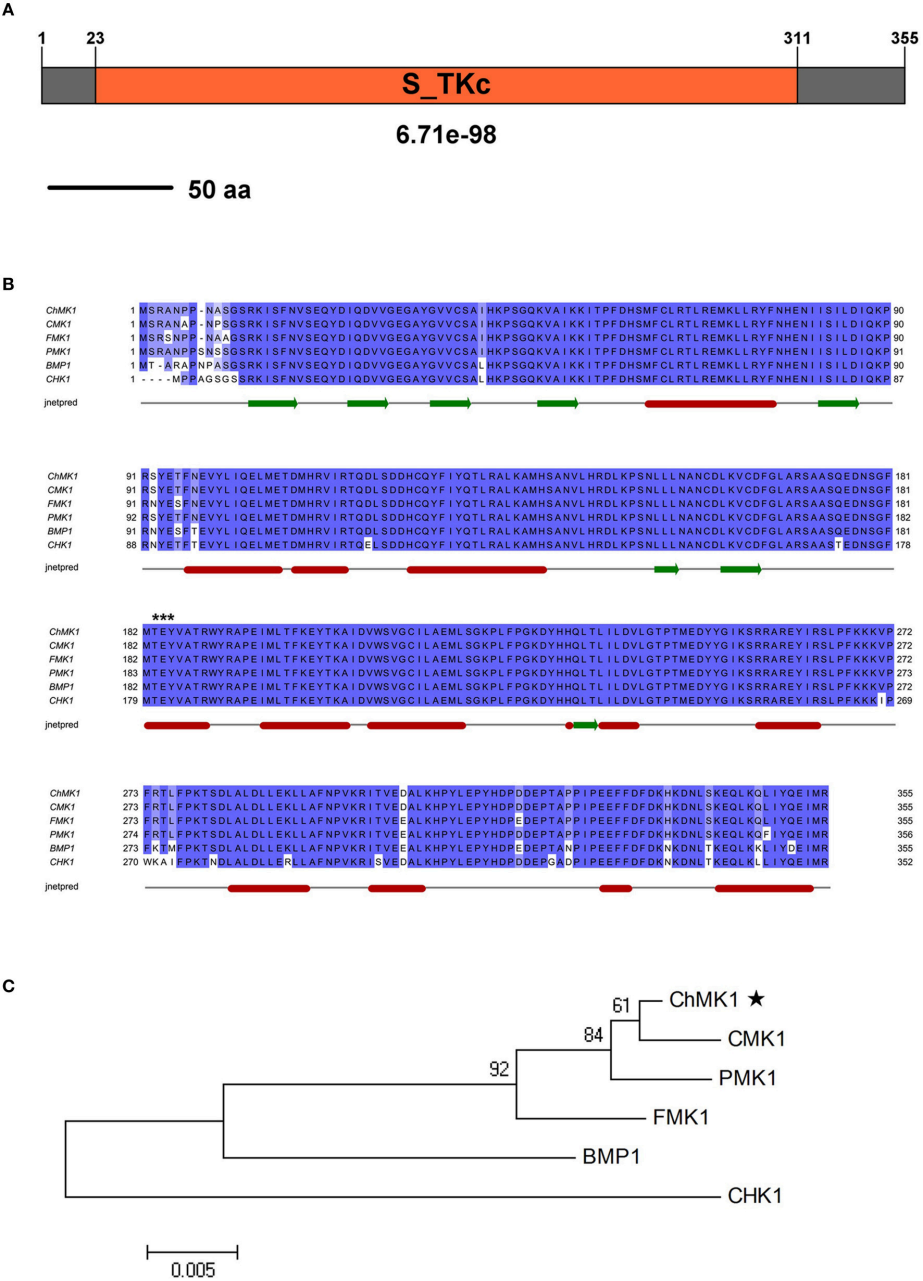
**Characterization of the *C. higginsianum* ChMK1 protein**. **(A)** The domain structure of *C. higginsianum* ChMK1 as annotated by *SMART MODE* (http://smart.embl-heidelberg.de/smart/change_mode.pl). **(B)** Alignment analysis of the amino acid sequences of ChMK1 (CH063_08490) and its homologs from other fungi using *Clustal X* program. PMK1: XP_003712175.1 (*M. oryzae*); CHK1: AF178977.1 (*C. heterostrophus*); BMP1: AF205375.1 (*B. cinerea*); FMK1: AAG01162.1 (*F. oxysporum*); CMK1: AAD50496.1 (*C. lagenarium*). The conserved kinase activation residues TEY are labeled with asterisks at the top of the alignment. The secondary structure prediction was completed with *Jnetpred* program. The green arrows indicate β-strands structure and the red sticks indicate α-helix structure. **(C)** Phylogenetic analysis of ChMK1 of *C. higginsianum* and its homologs from other fungi. The amino acid sequences were analyzed by MEGA version 4 with Unrooted Neighbor-joining algorithm. Bootstrap values were calculated from 1000 bootstrap replicates. Only bootstrap support values >50% are shown. The black star indicates ChMK1.

The *SMART MODE* (http://smart.embl-heidelberg.de/smart/change_mode.pl) analysis result indicates that CH063_08490 contains a Serine/Threonine protein kinases catalytic (S_TKc) domain (Figure 1A), which plays a key role in catalysis of protein phosphorylation. The BLAST searches of CH063_08490 for homologous sequences resulted in significant similarity with sequences from *M. oryzae* PMK1 (XP_003712175.1, *E*-value: 0.0, 98.9% identity), *Cochliobolus heterostrophus* CHK1 (AF178977.1, *E*-value: 0.0, 94% identity), *Botrytis cinerea* BMP1 (AF205375.1, *E*-value: 0.0, 94.6% identity), *F. oxysporum* FMK1 (AAG01162.1, *E*-value: 0.0, 98.3% identity) and *Colletotrichum lagenarium* CMK1 (AAD50496.1, *E*-value: 0.0, 99.4% identity) that match to the MAPK proteins. Sequence multiple alignment analysis of these homologs revealed significant conservation in length and amino acid composition (Figure 1B). Phylogenetic analysis indicated that CH063_08490 is closely related to CMK1 (Figure 1C), a MAPK in *C. lagenarium* involved in conidiation, appressorium formation, and pathogenicity (Takano et al., 2000).

In summary, the protein coded by *CH063_08490* resembles MAPK proteins, thus we named this gene as *ChMK1* derived from *C***. *h**igginsianum* *M**AP**K*.

### Gene disruption and complementation of *ChMK1*

**Figure 2:**
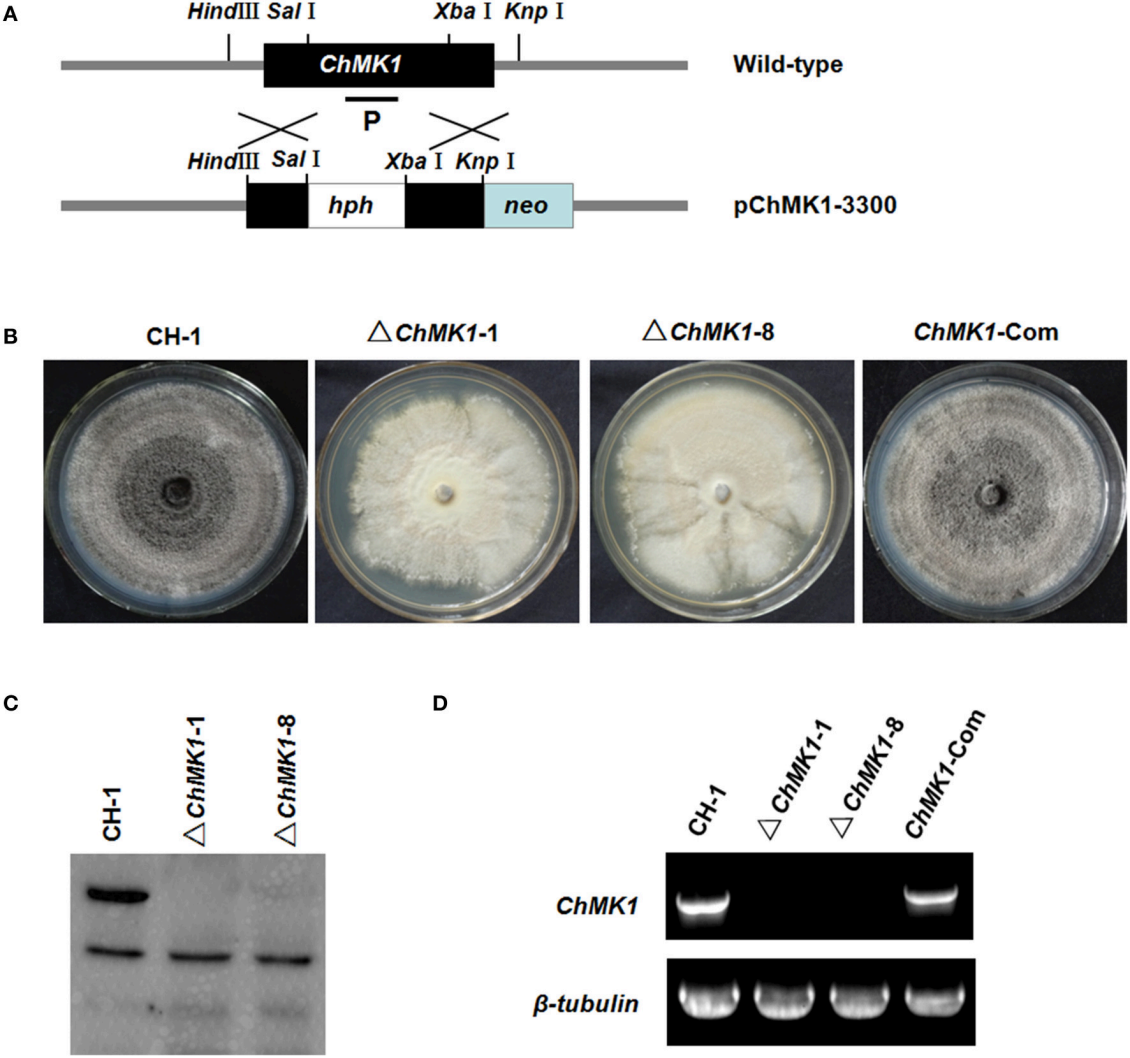
**Construction of replacement vectors and confirmation of *ChMK1* deletion mutants**. **(A)** Strategic map of the *ChMK1* replacement construct pChMK1-3300. Thick arrows indicate the orientations of *ChMK1* and hygromycin phosphotransferase (*hph*) gene. The *hph* gene cassette was cloned into the corresponding sites of vector pChMK1-3300 to replace the 1108-bp of the *ChMK1* ORF. **(B)** Colony morphologies of CH-1, ▵*ChMK1-1*, ▵*ChMK1-8*, and *ChMK1-Com* grown on PDA plate at 25°C for 15 d. **(C)** Southern blot analysis of mutants. Genomic DNA (15 μg per lane) of CH-1 (wild-type strain), ▵*ChMK1-1*, ▵*ChMK1-8*, and *ChMK1-Com* (*ChMK1* complementary transformant) were digested with *Sac*I. The same filter was hybridized with a probe corresponding to the *ChMK1* (P). **(D)** Total RNA samples isolated from mycelia of CH-1, ▵ *ChMK1-1*, ▵*ChMK1-8*, and *ChMK1-Com* were subjected to RT-PCR using *ChMK1* gene-specific primers 4F and 4R (Table 1). The RT-PCR product is a 560 bp fragment in CH-1 and *ChMK1-Com* as predicted, but is missing in the deletion mutants ▵*ChMK1-1*, ▵*ChMK1-8*.

For function study of *ChMK1* gene in *C. higginsianum*, we generated *ChMK1* deletion mutants. Deletion transformants were screened by growing on PDA containing hygromycin and further confirmed by Southern blot and RT-PCR (Figures 2C,D). Two deletion mutants ΔChMK1-1 and ΔChMK1-8, which had the marker inserted into regions other than the *ChMK1* gene, were selected for further analysis in this study. Furthermore, for complementation of the *ChMK1* deletion mutant, the complementary vector pChMK1-Com was transformed into deletion mutant ΔChMK1-1, and the complemented transformants ChMK1-Com, confirmed by RT-PCR was selected for further analysis (Figure 2D).

### *ChMK1* deletion transformant shows abnormal phenotype

**Figure 3:**
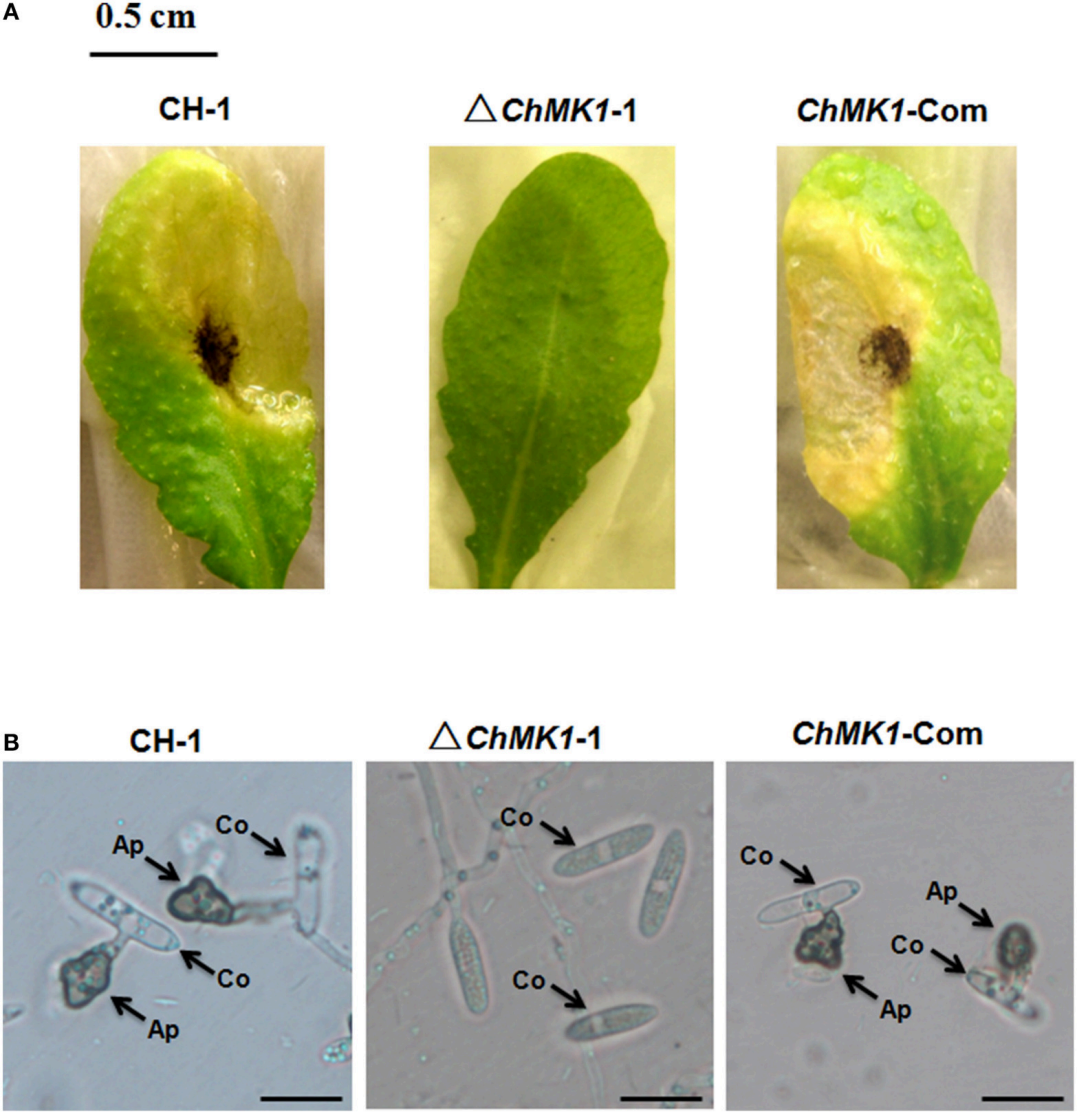
**Pathogenicity and appressoria formation**. **(A)** Disease symptoms on *A. thaliana* leaves caused by conidia suspension from CH-1, ▵*ChMK1-1*, and *ChMK1-Com*. Typical leaves were photographed 6 days after inoculation. Bar = 0.5 cm. **(B)** The development of appressoria on plastic microscopic coverslips at 25°C for 24 h by using Nikon Eclipse 80i microscope (Nikon, Tokyo, Japan), under bright-field model with 40 × fold magnification. Ap, appressoria; Co, conidia. Bars = 10 μm.

The infection assay of *ChMK1* deletion mutant was performed as described above. Conidia suspension of wild-type strain, *ChMK1* deletion strain ΔChMK1-1 and *ChMK1* complementary strain ChMK1-Com were inoculated on *Arabidopsis* leaves. At 6 dpi, the wild-type and ChMK1-Com strains could cause dark necrotic lesions (Figure 3A), whereas the *ChMK1* deletion transformant failed to cause any lesion on the leaves of *Arabidopsis* after inoculating ΔChMK1-1 mutants for 6 days (Figure 3A), which is similar with previous study that the MAPK mutant of *C. lagenarium* did not develop any lesions, even inoculated on wounded plants (Takano et al., 2000). These results indicate that *ChMK1* is essential for the pathogenicity of *C. higginsianum* on *Arabidopsis*.

The ChMK1 affects the melanin formation and growth rate of *C. higginsianum*. Compared with wild-type strain and *ChMK1* complementary strain, the *ChMK1* deletion transformant showed obvious albino colony (Figure 2B), reduction of conidiation (Table 2), and reduced growth rate on PDA (Table 2). Appressorium formation of the *ChMK1* deletion mutants on the hydrophobic surface (plastic coverslips) was investigated by microscopic observation. Conidia of the wild-type strain and *ChMK1* complementary strain could formed melanized appressoria on the plastic coverslips after incubation for 24 h, while *ChMK1* deletion mutant failed to differentiate into appressoria (Figure 3B). It concluded from these results that ChMK1 regulate appressorium formation, thus to affect the pathogenicity of *C. higginsianum*.

**Table 2:** **Comparison of growth rate and conidial production among *ChMK1* deletion mutants, complementary strain and wild-type strain**.

**Strains**	**Growth rate^a^ (mm/d)**	**Condiation^b^ (× 10^6^condia/plate)**
Wild-type	5.1 ± 0.2^A^	24.0 ± 2.0^A^
Δ*ChMK1-1*	2.8 ± 0.1^B^	6.4 ± 0.7^B^
Δ*ChMK1-8*	2.9 ± 0.1^B^	5.7 ± 0.6^B^
*ChMK1-Com*	5.2 ± 0.2^A^	26.5 ± 2.0^A^

a *Growth rate was detected by measuring the colony diameter of cultures incubated at 25°C for 7 days*.

b *Conidia produced by 15-days-old cultures and counted with haematocytometer. Different letters indicated statistically significant differences (P = 0.05). Means and standard errors were calculated from three replicates*.

### ChMK1 is significant for the maintenance of cell-wall integrity

**Figure 4:**
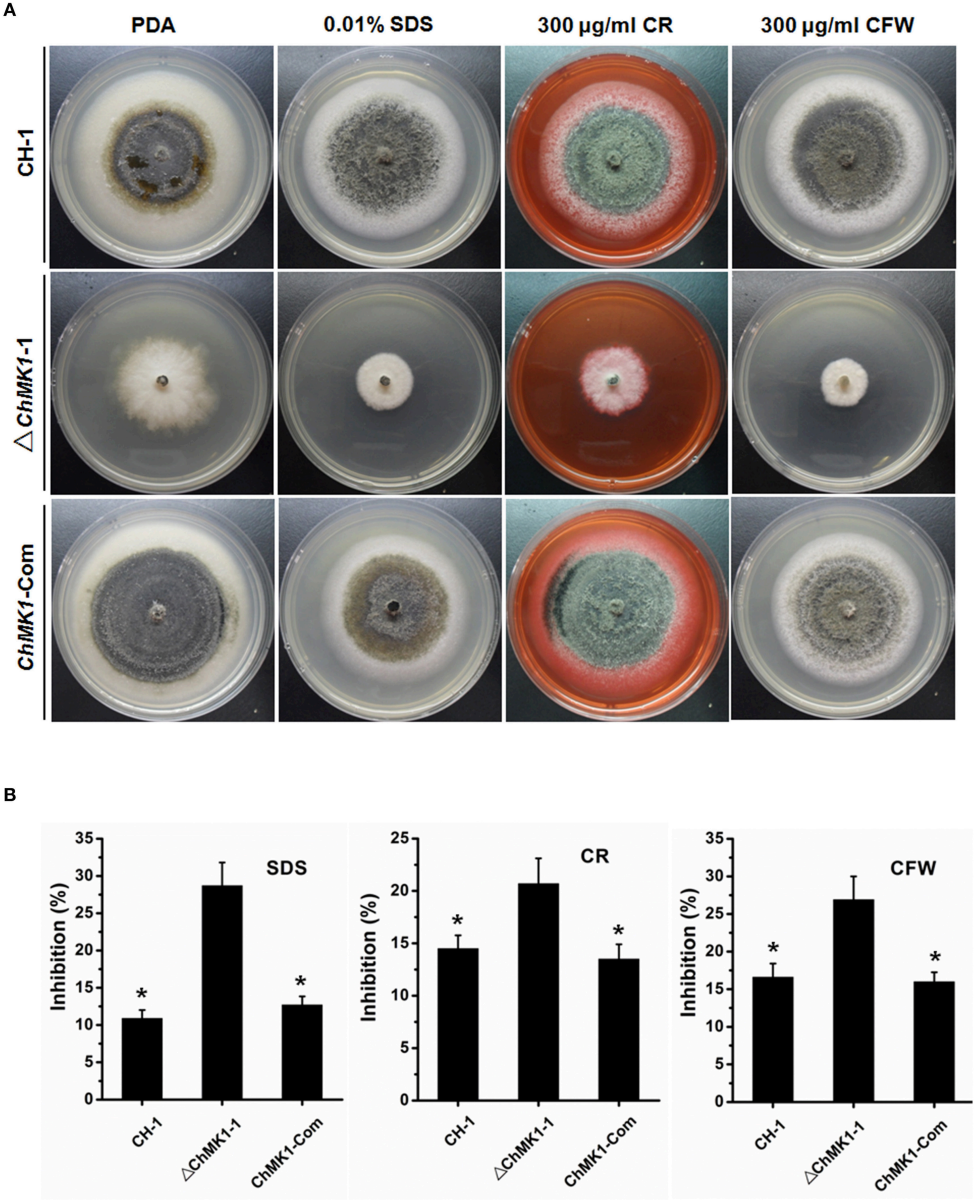
**Cell wall integrity assays of *C. higginsianum* to cell wall inhibitor**. **(A)** CH-1, ▵*ChMK1-1*, and *ChMK1-Com* treated with indicated cell wall inhibitor in PDA. Images were taken after 7 days of incubation on PDA with 0.01% SDS, 300 μg/ml Calcofluor White (CFW), and 300 μg/ml Congo Red (CR). **(B)** Inhibition of the radiated growth of CH-1, ▵*ChMK1-1*, and *ChMK1-Com* on the PDA with cell wall inhibitor. Means and standard errors were calculated from three replicates. Asterisks indicate statistical differences from the ▵*ChMK1-1* (*P* < 0.05).

Previous studies indicated that MAPK signal pathway contributed to cell wall integrity in multiple fungi (Jeon et al., 2008; Zeng et al., 2012; Ding et al., 2015), in our study, the sensitivity of wild-type strain, *ChMK1* complementary strain and *ChMK1* deletion transformant to three cell wall inhibitors was also tested. The results indicated that *ChMK1* deletion transformant was more sensitive to SDS, Calcofluor White (CFW) and Congo Red (CR) than wild-type strain and the complementary transformant (Figure 4A). Data showed that the inhibition of the growth rate of *ChMK1* deletion transformant were higher than that of wild-type strain and *ChMK1* complementary strain when cultured on PDA amended with those cell wall inhibitors respectively (Figure 4B).

### ChMK1 regulates the expression of melanin biosynthesis-associated genes

**Figure 5:**
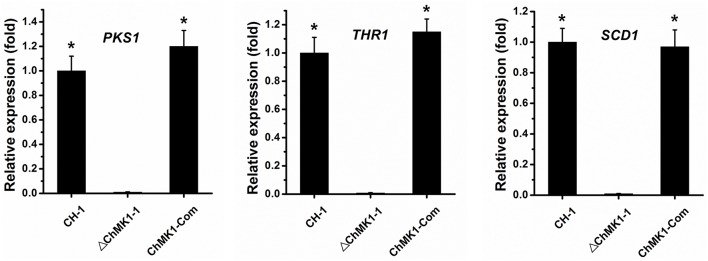
**The relative expression of *PKS1*, *THR1*, and *SCD1* in CH-1, ▵*ChMK1-1*, and *ChMK1-Com* cultured on PDA for 4 d**. The relative expression of target genes in CH-1 was set as level 1. Expression level of β*-tubulin* gene was used to normalize different samples. Bars represent means and standard deviations (three replications). Asterisks indicate statistical differences from the ▵*ChMK1-1* (*P* < 0.05).

Previous studies revealed a crucial role of MAPK signal pathway in melanin biosynthesis in several other fungi (Takano et al., 2000; Yago et al., 2011; Zeng et al., 2012). In our study, the *C. higginsianum ChMK1* deletion mutant was also unable to form melanized colony as wild-type, we speculated that the biosynthesis of dihydroxynaphthalene (DHN) melanin was interrupted. In order to verify this assumption, the expression level of three major melanin biosynthetic-associated genes, β*-ketoacyl synthase* (*PKS1, CH063_03518*), *trihydroxynaphthalene reductase* (*THR1, CH063_06688*), and *scytalone dehydratase* (*SCD1, CH063_08047*), were compared between *ChMK1* deletion mutant, complementary strain and wild-type strain using qRT-PCR. The results indicated that the expression level of these three genes in *ChMK1* deletion mutant mycelia was significantly reduced compared with that of the complementary and wild-type strains (Figure 5). Similar results were also obtained in other studies that the expression level of melanin biosynthetic-associated genes were decreased in MAPK mutants (Takano et al., 2000; Wei et al., 2016).

## Discussion

In this study, we characterized the Fus3/Kss1-related MAPK ChMK1 to assess the roles of MAPK pathways in the fungal pathogenesis of *C. higginsianum*. Gene deletion and complementary analysis of *ChMK1* demonstrated that this gene is essential for appressorium formation, pathogenicity, conidiation production, growth rate, maintenance of cell-wall integrity, and melanin formation in *C. higginsianum*.

In *F. oxysporum, C. lagenarium, M. oryzae*, and *Pyrenophora teres*, deletion mutants of Fus3/Kss1-related MAPK genes showed normal growth rates (Xu and Hamer, 1996; Takano et al., 2000; Di Pietro et al., 2001; Ruiz-Roldan et al., 2001; Luque et al., 2016), whereas the opposite was observed in *B. cinerea* and *Ustilago maydis* (Mayorga and Gold, 1999; Zheng et al., 2000). In our study, the growth rate of *ChMK1* deletion mutants was significantly decreased compared to wild type strain (Table 2), supporting the diverse functions of Fus3/Kss1-related MAPKs in different plant fungal pathogens. Furthermore, the Fus3/Kss1-related MAPK signal pathway from several plant pathogen fungi plays significant roles in conidiation, appressorium formation, and fungal pathogenicity (Mayorga and Gold, 1999; Ruiz-Roldan et al., 2001; Zhao et al., 2007; Miguel-Rojas and Hera, 2016; Zhang et al., 2016). In this study, the albinistic *ChMK1* deletion mutant also showed a significant reduction in conidiation production (Table 2) and defects in appressorium formation, thus losing pathogenicity (Figure 3). These results indicate the conserved roles of Fus3/Kss1-related MAPKs in phytopathogenic fungi.

SLT2-type MAPKs, another type of MAPKs, are involved in cell wall integrity and stress tolerance in multiple fungi and *Phytophthora* species (Gao et al., 2015). In *Coniothyrium minitans*, CmSlt2 was involved in conidiation, cell wall integrity, and mycoparasitism, targeted disruption of *CmSlt2* led to hypersensitivity to cell wall-degrading enzymes and cell wall inhibitors caffeine, CFW and CR (Zeng et al., 2012). In *F. oxysporum*, disruption of the MAPK *FoSlt2* gene resulted in increased sensitivity to H_2_O_2_ and cell wall inhibitors CR and CFW (Ding et al., 2015). In *M. oryzae*, MoMck1–MoMkk1–MoMps1 MAPK pathway contributed to regulate cell wall integrity (Yin et al., 2016). Moreover, silencing of *PsMPK1* in *P. sojae* caused increased hypersensitivity to cell wall-degrading enzymes cellulase and lysing enzyme (Li et al., 2014). Similar results were obtained in *A. alternata*, since the *AaSLT2* mutants also displayed hypersensitivity to cell wall-degrading enzymes, CFW and CR (Yago et al., 2011). Nevertheless, until recently, there has been little experimental evidence for the contribution of Fus3/Kss1-related MAPKs to cell wall integrity in fungi. In this study, our results firstly demonstrated that the Fus3/Kss1-related MAPK ChMK1 from *C. higginsianum* also plays a significant role in cell wall integrity, and deletion of *ChMK1* cause increased hypersensitivity to cell wall inhibitors (Figure 4).

Furthermore, the deletion mutants of *ChMK1* showed obviously albino colony (Figure 2B). Since DHN melanin is required for melanization, which is essential for appressorial functions and virulence in *Colletotrichum* species (Rasmussen and Hanau, 1989; Lin et al., 2012), we speculated that the biosynthesis of DHN melanin was interrupted in the deletion mutant of *ChMK1*. The qRT-PCR results in this study demonstrated that the expression levels of *PKS1, THR1*, and *SCD1*, the major DHN melanin biosynthetic-associated genes (Takano et al., 1997), decreased significantly in *ChMK1* deletion mutant when compared to wild-type strain (Figure 5), which was similar with other studies that the expression level of melanin biosynthetic-associated genes decreased in MAPK mutants (Takano et al., 2000; Wei et al., 2016), indicating that ChMK1 is involved in the biosynthesis of DHN melanin by regulating the expression of DHN melanin biosynthetic-associated genes.

Most fungal pathogens contain three MAPKs that are orthologs of the *S. cerevisiae* Fus3/Kss1, Slt2, and Hog1 MAPK. Recent study demonstrated that Fus3/Kss1, Slt2, and Hog1 MAPKs have distinct and complementary roles, and the positive and negative crosstalk between three MAPK pathways regulates stress adaptation, development and virulence in *F. oxysporum* (Luque et al., 2016). In *M. oryzae*, it was reported that the feedback between the cAMP and MAPK signaling pathways regulate appressorium morphogenesis and plant infection (Zhou et al., 2012). In *C. lagenarium*, Cmk1 MAPK cooperated with cAMP-PKA signaling pathway to regulate germination, appressorium formation and infectious growth, and mutation of these genes led to similar defects in germination, appressorium formation, and infectious growth (Takano et al., 2000; Yamauchi et al., 2004). In *B. cinerea*, Nox-, calcium-, and MAPK-signaling cascades incorporated with RasGAP scaffold protein BcIqg1 to regulate several developmental processes and virulence (Marschall and Tudzynski, 2016). Similar results were also obtained in mycoparasite *C. minitans* that MAPK cascade and Nox complex signal pathway are cross-linked and essential for pathogenicity, melanin synthesis and conidiation (Wei et al., 2016). In our study, deletion of *ChMK1* resulted in decreased growth rate, defects in virulence, and appressorial formation, hypersensitivity to cell wall inhibitors, reduced conidiation, and albinistic colony. These phenotype of *ChMK1* mutant are similar with that of the mutants of other signaling pathway in other fungi (Zhou et al., 2012; Luque et al., 2016; Marschall and Tudzynski, 2016; Wei et al., 2016). Thus, it was speculated that *C. higginsianum* MAPK cascade also cross-linked with other signal pathways possibly to regulate multiple physiological processes in *C. higginsianum*.

Briefly, we have analyzed the functions of ChMK1 in appressorium formation, pathogenicity, growth rate, conidiation and melanin production in *C. higginsianum*. Moreover, we also firstly reported that the Fus3/Kss1-related MAPK is involved in the maintenance of cell-wall integrity. The results described above will enhance our understanding of the mechanism underlying *A. thaliana*–*C. higginsianum* interaction and will facilitate the efficient control of cruciferous crops anthracnose disease.

## Conclusion

The Fus3/Kss1-related MAPK ChMK1 was experimentally confirmed to be essential to appressorium formation, pathogenicity, growth rate, conidiation production, and melanin formation in *C. higginsianum*. Furthermore, our results also firstly showed the involvement of Fus3/Kss1-related MAPK in cell wall integrity, indicating that ChMK1 plays diverse and essential roles in this fungus.

## Author contributions

Conceived and designed the experiments: WW, WZ. Performed the experiments: WW, WZ. Analyzed the experiment data: WW, YX, WZ, NW, GY, FP. Contributed reagents/materials/analysis tools: WW, WZ. Wrote the paper: WW, WZ. All authors have read and approve the final manuscript.

### Conflict of interest statement

The authors declare that the research was conducted in the absence of any commercial or financial relationships that could be construed as a potential conflict of interest.

## Supplementary material

Figure S1 **The vector map of pChMK1-3300**.
