# Supplementary figures and images for: Colletotrichum higginsianum Mitogen-Activated Protein Kinase ChMK1: Role in Growth, Cell Wall Integrity, Colony Melanization, and Pathogenicity
Source: Front Microbiol. 2016 Aug 3;7:1212. doi: 10.3389/fmicb.2016.01212 (PMC4971432; doi:10.3389/fmicb.2016.01212)

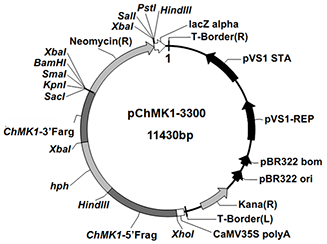

Supplement: Figure S1 — The vector map of pChMK1-3300. [file Image1.TIF]
